# Importance of ECG in the Diagnosis of Acute Pericarditis and Myocardial Infarction: A Review Article

**DOI:** 10.7759/cureus.30633

**Published:** 2022-10-24

**Authors:** Aditya K Sarda, Preeti Thute

**Affiliations:** 1 Medicine and Surgery, Jawaharlal Nehru Medical College, Datta Meghe Institute of Medical Sciences, Wardha, IND; 2 Anatomy, Jawaharlal Nehru Medical College, Datta Meghe Institute of Medical Sciences, Wardha, IND

**Keywords:** pr segment and t wave, q wave, st segment, electrocardiograph, acute pericarditis, acute myocardial infarction

## Abstract

A promising scientific field is health monitoring and associated technology. A standard testing method to evaluate and identify heart issues is the electrocardiogram (E.C.G.) and diagnose cardiovascular diseases (CVDs). E.C.G. monitoring technologies are becoming more and more prevalent in publications at an exponential rate. E.C.G. is the most crucial tool for screening cardiology and other medical specialities. Twelve leads can be recorded by traditional E.C.G. equipment, while current E.C.G. systems allow for extra leads also with fewer electrodes. Furthermore, “smart” gadgets allow patients to take an E.C.G. at residence. Presenting different ischemia-related symptoms on the E.C.G. by the most recent recommendations. Presentation of contemporary E.C.G. systems and their possible benefit in identifying ischemia-related E.C.G. symptoms based on recent study findings. The identification of ischemia E.C.G. abnormalities can be facilitated and optimised by current E.C.G. systems using vector-based electrocardiography. Although they can be effective for documenting transient E.C.G. abnormalities, especially inside the S.T. segment, smart non-vector-based devices for patients are primarily beneficial for the diagnosis of arrhythmias and cannot substitute the 12-lead E.C.G. for the diagnosis of ischemia. The electrocardiogram (E.C.G.) is inexpensive and easily accessible, but because of its alleged limited specificity, its utility as a screening tool for early detection of athletes with a cardiac condition in danger of immediate cardiac death is contentious. The interpreting parameters have been continuously evolving over the past 10 years as various efforts have been made to better the separation between healthy and pathological E.C.G. abnormalities in athletes. Electrocardiographic abnormalities that are unrelated to cardiac electrical activity are known as electrocardiographic artefacts. E.C.G. elements, including the baseline and waves, can become altered as a result of artefacts.

## Introduction and background

Acute myocardial infarction (MI) is a word coined for a match of coronary assault, which is caused by the deposition of plaques or cholesterol in the inner surface of the coronary arteries resulting in decreased blood flow to the heart and scarring the cardiac muscle mass due to insufficient oxygen supply [1]. Though MI primarily affects patients over the age of 45, it can also affect young men or women. Fortunately, patients under 45 years old do not often experience its occurrence [2]. Cardiovascular disease has a fatality rate that is greater than twice as high as cancer in the U.S., making it the most common cause of death, and over half of these vascular fatalities are attributable to acute myocardial attack/infarction. The discussion of acute attack/MI patient management during and after hospitalisation places a focus on primary and secondary prevention, patient autonomy, and decision-making. An assessment of the future directions for treating acute MI is also included [3]. Viral infections are frequently linked to acute pericarditis. The typical symptom of infection is chest discomfort, which manifests in conjunction with or shortly after the indications of infection [4]. About 5% of patients who are brought to the emergency medicine department with thoracic discomfort not related to an acute myocardial attack/infarction are found to have acute pericarditis, which is referred to as pericardial inflammation. Most typically, it affects males between the ages of 20 and 50 [5]. The most prevalent symptom in people with an acute MI is chest pain, which is commonly described as tightness in the chest, pressure, or a squeezed feeling [6].

The majority of MI with non-obstructive coronary artery (M.I.N.O.C.A.) patients who experience an acute myocardial attack/infarction with S.T. segment rise from the baseline typically have a pre-existing coronary or myocardial aetiology, most frequently plaque disruption or myocarditis [7]. Acute pericarditis, an acute inflammatory sickness that affects the pericardium, can result from several different illnesses (both non-infectious and infectious). The diagnosis is often made based on symptoms (chest discomfort, difficulty breathing), electrocardiogram (E.C.G.) abnormalities (S.T. amplification), physical assessment (pericardial friction rubbing), and elevation of cardiac biomarkers. It could take either alone or in tandem with an involved inflammatory disorder. Acute pericarditis and acute myocarditis can cohabit in routine clinical practice due to their convergent aetiologies [8]. Inflammation of the pericardium (heart covering), also known as acute pericarditis, can result in cardiac involvement and pericardial effusion (peri myocarditis). Systemic inflammatory rheumatic illnesses can cause pericarditis. However, it can also represent a distinct disease entity [9]. Non-S.T.-segment elevation MI (NSTEMI) and S.T.-segment elevation MI (STEMI), in which S.T.-segment elevation is observed on the E.C.G. graph, are the two kinds of acute MI [10]. A frequent condition with numerous causes that can manifest in both primary and secondary care settings is pericarditis. The sampling and analysis of pericardial fluid have been improved by new diagnostic techniques, which enable thorough cause characterisation. Despite these developments, pericarditis still most frequently arises from idiopathic causes; they include radiation therapy, heart surgery, and percutaneous procedures. Non-steroidal anti-inflammatory drugs continue to be the primary choice of treatment for simple instances of pericarditis because it typically has a self-limiting course. Integrating new imaging techniques makes it easier to accurately identify and treat complications like acute pericardial effusion or constriction. An echo-guided percutaneous technique can be used to safely treat the majority of pericardial effusions. In most situations, pericardiectomy relieves symptoms and is still the only effective treatment for constrictive pericarditis [11]. A thorough physical examination, an E.C.G., an echocardiogram, blood work, and still a chest x-ray is frequently necessary to make the clinical finding of acute pericarditis. Acute pericarditis and S.T.-elevated myocardial infarct share comparable E.C.G. features, making the distinction between the two frequently challenging (STEMI) (S.T. segment change) [12].

## Review

ST-segment elevation

Cases of S.T.-segment upliftment from baseline in MI with S.T.-segment upliftment from the baseline in the anterior and anteroposterior territories, and less frequently, concurrent S.T.-elevations in the anterior and inferior electrocardiography leads. To identify this cardiac emergency, which frequently requires urgent coronary revascularisation, accurate interpretation of the E.C.G. is essential. Additionally, E.C.G. improves prognostic value and helps localise the artery connected to the infarct [13]. Because of epidemiologic information and arteriographic correlative investigations, the S.T.-segment reaction to exercise is frequently utilised to identify people with ischemic heart disease [14]. Abrupt S.T.-segment rise (RT checkbox indication), horizontal or upwards S.T.-segment convexity, lead III (three/3) S.T.-segment rise greater than lead II (two/2) S.T.-segment elevation and lead III > II To assess the strength of the link, we utilised the incidence rate with such a 95% confidence interval (CI) [15]. Every year, approximately seven million people worldwide are identified with ACS. In STEMI patients, mortality is reduced by cardiac catheter and PCI within one or 20 minutes of presentation; fibrinolytic therapy is only administered to those who are unable to undergo prompt PCI. Low death rates are associated with rapid invasive coronary angiography (RICA), followed by interventional or surgical revascularisation, in high-risk NSTE-ACS patients [16]. The 12-lead E.C.G. continues to be the mainstay of early STEMI diagnosis, and it also serves as the key indicator for S.T.-elevation MI patients requiring emergency reperfusion treatment. These are characterised as S.T. rise MI mimics and includes, for example, left ventricular dilatation, acute pericarditis, and benign early repolarization. In rare clinical circumstances, a patient's E.C.G. may mimic S.T. elevation MI yet exhibit S.T.-segment elevated from a non-coronary-based illness [17]. The ability of pericarditis and early repolarization syndrome (ERS) and takotsubo cardiomyopathy to resemble S.T. elevation MI is well documented (STEMI). Acute chest discomfort and an S.T. rise of a fraction of 1 mV were present in all individuals. For the existence of S.T.-segment convexity, each E.C.G. was evaluated [18]. The physical sign of acute pericarditis is the pleural frictional rub, which is often audible around the lower lateral sternal border. An easy tool for diagnosing acute pericarditis is the E.C.G. Diffuse concave-upwards S.T.-segment rise and, in rare instances, P.R.-segment depression are common E.C.G. abnormalities. Early repolarisation and acute MI both have E.C.G. abnormalities that resemble acute pericarditis [19].

Q-wave

Q-waves may indicate acute MI when seen on an E.C.G. There is no distinction between transmural and non-transmural infarction in the E.C.G. alterations. On the other hand, a Q-wave's existence or absence corresponds with several features of a patient's post-MI clinical history and hence has predictive significance. Congestive heart failure is more likely to worsen Q-wave infarctions while they are being treated in the hospital [20]. Congestive heart failure was more likely to occur later in participants who had their first Q-wave infarction, although coronary insufficiency was more common in participants who had their first non-Q-wave infarction [21]. A chest pain attack caused aberrant Q waves to arise in Leads V2 and V3, but they quickly vanished after the discomfort subsided. Although MI could not be ruled out, the serial electrocardiographic alterations and the clinical history were consistent with angina or coronary insufficiency. We think that the myocardium's transient ischemia was what caused these Q waves [22]. Q waves and amplified CKMB activity are the presentations of pericarditis, which must be separated from MI brought on by coronary artery disease [23]. Infarction-associated pericarditis's pericardial effusion and elevated extravascular lung water were not brought on by left ventricular failure but rather by other causes that showed a greater infarct [24]. Acute MI typically begins with aberrant Q waves, which are frequently seen. However, there is not any evidence that abnormal Q waves following thrombolytic treatment have a huge negative impact on the ability to reduce infarct size [25]. Although reversible Q.R.S. complex alterations linked to an S.T.-segment shift are a hallmark of acute myocardial ischemia (AMI), these changes have yet to be comprehensively examined in a significant patient cohort. Over the past four years, a deliberate hunt for E.C.Gs with confirmed reversible Q.R.S. alterations connected to any acute injury type has been made [26].

PR segment depression

Significant P.R.-segment depressed > or = 1.2 millimetres in the inferior leads was linked to an uncomfortable hospital stay and a subpar short-term prognosis in acute inferior MI. These individuals had a high chance of developing heart free-wall rupture, atrioventricular block, and supraventricular arrhythmias [27]. When there were aberrant P waveforms or P.R. dislocation in either lead, the duration of stay was longer. Patients with an aberrant P-wave had a higher likelihood of having left main cardiovascular disease. Higher one-month and 12-month death rates were linked to aberrant P-wave shapes in any lead (OR 3.09 [1.35-7.05] and 5.33 [2.74-10.36], respectively). Additionally, increased one month (OR 2.33; 1.03-5.28) and 12-month mortality were linked to P.R. displacement in just about any lead. P.R. deficiency in II, III, and AFV, as well as elevation in AVR or AVL and P.R. depression in the precordial leads, were all linked to an increase in one-year fatality (OR 12.49). (5.2-30.0). When age, ejection fraction, peak biomarker troponin I, and left the primary illness were taken into account, it was shown that P.R. displacement in either lead was linked to higher one-year mortality rates. When present in 31% of STEMI patients, P.R. segment relocation in any lead independently predicted 12-month mortality [28]. The distinctive E.C.G. signs of acute pericarditis are P.R.-segment distress (found rarely), multi-lead S.T. segment rise, and S.T.-segment distress in the lead named aVR [29].

Reciprocal T-wave changes

The behaviour of main T-wave abnormalities following exercise does not modify the interpretation of the ischemic alterations, according to exercise-induced T-wave normalisation studies. T-wave abnormalities are typically non-specific. However, isoproterenol treatment prevents post-MI T-wave modifications while treating a variety of functional and neurogenic T-wave abnormalities [30]. A thallium-201 scan was positive in 49 of 52 myocardium segments with controlled T impulses and 43 of 45 individuals (95%) overall. When the regions of engagement were linked to thallium scanning and T-wave changes, 76% of lead aV's T-wave normalisation was related to positive inferior-posterior scans, and 77% of V1 and V5's T-wave normalisation was connected with positive anterior-septal scans [31]. Only after comparing the precordial leads to the baseline, E.C.G. was the little T-wave alterations visible. The goal of the current investigation was to see if reciprocal alterations could occur in place of or in addition to any other symptoms of AMI [32]. The normalisation method for normalisation could include producing an isoelectric-electric S.T. segment and upright T-wave by adding algebraically the immediate ischemia-induced S.T. segment and T-wave magnitude in addition to the pre-existing S.T. segment lowering, and T-wave inversion. Recordings of reciprocal changes, such as MI, are possible [33].

T-wave inversion

Inverted T waves provide diverse anatomical lead groups with varied prognostic data. A higher risk of CHD is associated with a T-wave reversal in the lateral and anterior lead groups, and an increased risk of death is associated with lateral T-wave reversal. It was discovered that the inferior lead group's inverted T-wave was a harmless occurrence [34]. It is challenging to interpret the T waveform on an E.C.G. It relies on changes to cell membranes because it represents ventricular repolarisation. It is influenced by several physiological, central nervous system, and physical health aspects. It is the most unstable part of the QRST complex and the most sensitive sickness indicator. The huge intensification of the T waveform, which is monstrously blown out, typically inverted, and covers an area many times that of the Q.R.S. complex, is one of the most striking and least understood oddities. Heart illness has frequently been brought to light, particularly whenever the T waveforms are inverted [35]. Conventionally thin and symmetric inverted T waves are brought on by myocardial ischemia. The isoelectric S.T. section is frequently concave when a T-waveform inversion (TWI) is associated with acute coronary syndrome (ACS), and this is followed by a powerful symmetric downstroke [36]. Patients having anterior STEMI who simultaneously exhibited inverted T waves inside the leads and S.T. rise might be identified by an accurate indicator of spontaneous reperfusion of an infarct-related artery on the E.C.G. [37]. Value of the recently found T-wave inversion as a predictor in patients who have undergone first percutaneous coronary interventions and have elevated S.T.-segment MI (PCI). New T-wave inversion is the beginning of T-wave inversion during the percutaneous coronary intervention (PCI) without the presence of negative T-waves only on the displayed E.C.G. Following the first PCI, a recently created T-wave inversion was linked to a positive long-term result [38].

Table 1 reflects changes in E.C.G. for differentiating acute pericarditis and acute MI based on the five features that include depression in the P.R. segment, changes in Q-wave, elevation of S.T. segment, inverted changes of T-wave and Inversion of T-wave.

**Table 1:** Differentiating between acute pericarditis and acute myocardial infarction

Sr. No.	ECG Features	Acute Pericarditis	Acute Myocardial Infarction
1.	Depression in the PR Segment	Can be seen in various patients	Very rare to be noticed
2.	Changes in Q-Wave	Not seen in patients with the above condition	Seen in patient with above condition
3.	Elevation of ST-Segment	Presented with concave elevation	Presented with convex elevation
4.	Inverted changes of T- Wave	Not presented in patients with the above condition	Seen in patients with the above condition
5.	Inversion of T-Wave	Seen in ECG once when the changes of ST-Segment are normalized	Seen in ECG once when the changes of ST-Segment are normalized

## Conclusions

Normal E.C.G. has 12 leads which are used to measure the cardiac abnormalities in a person. There is a baseline in E.C.G., P-wave, Q.R.S. complex, and T-wave. The basic analysis of acute pericarditis and acute MI can be diagnosed on the E.C.G.

It can also be differentiated based on some factors in E.C.G. The S.T.-segment upliftment denotes the rise of the S.T.-segment portion from the baseline. If the elevation of the S.T. segment is convex at the top of the elevated line, it is diagnosed as an acute MI, which is further studied in the limb for precisely conforming to the artery that is blocked, while in the case of the S.T. segment elevation if the elevated segment forms the concave curve at the top of the elevation, it is diagnosed as acute pericarditis. Q-wave is the starting negative bend of the rise to the Q.R.S. complex. Q-wave is the representation of the depolarisation of the ventricular in E.C.G. Q-wave is absent in the E.C.G. of patients suffering from acute pericarditis, while the Q-wave is present with no or minimal changes in the patient suffering from sudden MI. The P.R. segment is the amount of time that passes between the atrium and ventricle depolarising. One of the most crucial elements in the diagnosis of acute pericarditis, along with depression, is the P.R. segment in the P.R. segment is the most widely presented feature in E.C.G. of the patient who is suffering from acute pericarditis, but on the other hand, the P.R.-segment depression is one of the rarest phenomena presented by the patients of acute MI. T-wave represents ventricular myocardium repolarisation. Reciprocal T-wave is a very abnormal change of T-wave in the aVL, which is very typically transmural. These reciprocal T-wave changes are usually not presented in the E.C.G. of the patient with acute pericarditis. At the same time, the reciprocal T-wave changes are considered one of the most important changes in E.C.G. for the diagnosis of acute MI in patients. Inversion of T-wave is well-defined as the negative movement of T-wave in more than two contagious leads, excluding the leads like III, aVR, and V1. This T-wave cannot be considered an important factor for the differentiation of acute pericarditis and acute MI, as it is seen in both patients suffering from the above disorders. But the important thing is that it is seen only after the S.T.-segment upliftment is normalised in the E.C.G. of the patient suffering from the above disorder.
